# Differential neuropeptide modulation of premotor and motor neurons in the lobster cardiac ganglion

**DOI:** 10.1152/jn.00089.2020

**Published:** 2020-08-05

**Authors:** Emily R. Oleisky, Meredith E. Stanhope, J. Joe Hull, Andrew E. Christie, Patsy S. Dickinson

**Affiliations:** ^1^Department of Biology, Bowdoin College, Brunswick, Maine; ^2^Pest Management and Biocontrol Research Unit, US Arid Land Agricultural Research Center, USDA Agricultural Research Services, Maricopa, Arizona; ^3^Békésy Laboratory of Neurobiology, Pacific Biosciences Research Center, School of Ocean and Earth Science and Technology, University of Hawaii at Manoa, Honolulu, Hawaii

**Keywords:** cardiac ganglion, central pattern generator, *Homarus americanus*, myosuppressin, neuropeptide

## Abstract

The American lobster, *Homarus americanus*, cardiac neuromuscular system is controlled by the cardiac ganglion (CG), a central pattern generator consisting of four premotor and five motor neurons. Here, we show that the premotor and motor neurons can establish independent bursting patterns when decoupled by a physical ligature. We also show that mRNA encoding myosuppressin, a cardioactive neuropeptide, is produced within the CG. We thus asked whether myosuppressin modulates the decoupled premotor and motor neurons, and if so, how this modulation might underlie the role(s) that these neurons play in myosuppressin’s effects on ganglionic output. Although myosuppressin exerted dose-dependent effects on burst frequency and duration in both premotor and motor neurons in the intact CG, its effects on the ligatured ganglion were more complex, with different effects and thresholds on the two types of neurons. These data suggest that the motor neurons are more important in determining the changes in frequency of the CG elicited by low concentrations of myosuppressin, whereas the premotor neurons have a greater impact on changes elicited in burst duration. A single putative myosuppressin receptor (MSR-I) was previously described from the *Homarus* nervous system. We identified four additional putative MSRs (MSR-II–V) and investigated their individual distributions in the CG premotor and motor neurons using RT-PCR. Transcripts for only three receptors (MSR-II–IV) were amplified from the CG. Potential differential distributions of the receptors were observed between the premotor and motor neurons; these differences may contribute to the distinct physiological responses of the two neuron types to myosuppressin.

**NEW & NOTEWORTHY** Premotor and motor neurons of the *Homarus americanus* cardiac ganglion (CG) are normally electrically and chemically coupled, and generate rhythmic bursting that drives cardiac contractions; we show that they can establish independent bursting patterns when physically decoupled by a ligature. The neuropeptide myosuppressin modulates different aspects of the bursting pattern in these neuron types to determine the overall modulation of the intact CG. Differential distribution of myosuppressin receptors may underlie the observed responses to myosuppressin.

## INTRODUCTION

Flexibility in neuronal output underlies the ability of pattern-generating networks to elicit a wide array of rhythmic movements (e.g., breathing, locomotion); such flexibility is frequently achieved by the action of neuromodulators (Delcomyn 1980; Marder and Calabrese 1996). Both circulating hormones and locally released neuromodulators have been shown to act on neural networks to elicit the physiological changes that lead to changes in behavioral output, although they generally do so at different concentrations (Dickinson et al. 2015, 2019a; Messinger et al. 2005). While a wide range of molecules can serve as neuromodulators, peptides comprise the largest and most diverse group (Brezina 2010; Briggman and Kristan 2008; Christie et al. 2010a).

**Fig. 1.**
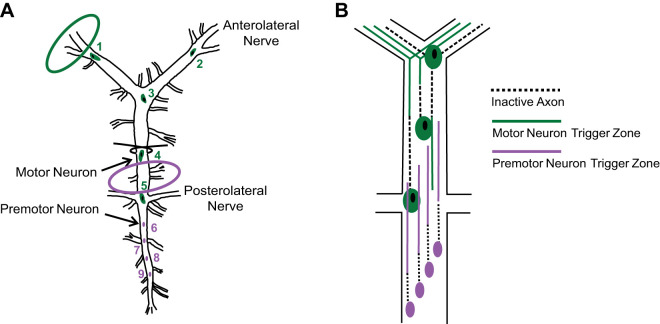
Schematic diagram of the cardiac ganglion (CG) of the American lobster, *Homarus americanus*. *A*: organization of the cardiac ganglion with ligature and petroleum jelly well placement. Four small premotor neurons located in the posterior trunk of the cardiac ganglion are electrically and chemically coupled to the five large motor neurons, which are located in the anterior portion of the cardiac ganglion. All nine neurons are electrically and chemically coupled. Green indicates motor neurons and purple indicates premotor neurons. Green and purple ovals indicate the location of recording sites. Site of the ligature is indicated by thread loop. *B*: diagram of physiologically determined anatomy (Hartline 1967) of the trigger zone locations within the trunk of the CG.

The decapod crustacean cardiac neuromuscular system is a model for understanding the modulatory control of rhythmic motor behavior (Cooke 2002). This simple central pattern generator (CPG)-effector system is composed of the cardiac ganglion (CG), i.e., the CPG, and the heart musculature, i.e., the effector system. In the American lobster, *Homarus americanus*, the CG consists of nine neurons (Fig. 1*A*): four small, posteriorly positioned premotor neurons (alternatively termed the small cells or pacemaker neurons) and five large, anteriorly located motor neurons (also referred to as the large cells). These two types of neurons are both electrically and chemically coupled and exhibit spontaneous in-phase bursting activity (Cooke 2002; Williams et al. 2013). The premotor neurons have classically been viewed as driving rhythmic activity in the CG by synapsing onto and promoting burst activity in motor cells (Hartline 1967). The motor neurons send feedback to the premotor neurons, and endogenous driver potentials contribute to the regulation of burst frequency (Berlind 1989; Mayeri 1973). The collective actions of the two neuron types produce bursts of action potentials that drive heart contractions (Cooke 2002). We report here that the premotor and motor neurons of the lobster CG can establish independent bursting patterns when physically decoupled by a ligature. Since peptides have been shown to alter the crustacean CPG bursting behavior at both the level of the isolated ganglion and the periphery of the cardiac neuromuscular system (Dickinson et al. 2007; Fort et al. 2007a, 2007b; Stevens et al. 2009), we asked whether the separated neuronal types were independently modulated, and if so, what roles each neuron type might play in the modulation of the pattern as a whole.

Myosuppressin (pQDLDHVFLRFamide), a highly conserved decapod neuropeptide (Stemmler et al. 2007), has been shown to act at multiple sites within the cardiac neuromuscular system of the lobster. In the whole heart, myosuppressin (10^−7^ M) decreases heart contraction frequency and causes an initial decrease in contraction amplitude followed by a large increase in amplitude. In the isolated CG, myosuppressin elicits a decrease in burst frequency and an increase in burst duration, with the threshold for effects at ~10^−7^ M. When motor input was removed from the heart and an electrode was used to deliver an electrical stimulus mimicking the CG bursting pattern, amplitudes of cardiac contractions increased in the presence of 10^−7^ M myosuppressin, suggesting that myosuppressin acts at the neuromuscular junction or muscle as well as in the CG itself to elicit whole heart responses (Stevens et al. 2009).

In this study, we investigated whether myosuppressin was capable of independently modulating the premotor and motor neurons of the CG, and if so, how such modulation might underlie the role of these neurons in the coordinated motor pattern. Because many of the effects of myosuppressin recorded previously and in the present study are evident only at concentrations that are consistent with local rather than hormonal release (i.e., 10^−6^ M rather than 10^−7^ to 10^−9^ M; Dickinson et al. 2015, 2019a; Messinger et al. 2005), we asked whether myosuppressin is likely to be present within the neurons of the CG. In support of this, we found putative myosuppressin-encoding transcripts expressed in the neurons of the CG. Finally, to elucidate mechanisms that may underlie the differential effects on the two neuronal types in the CG, we asked whether the CG expressed more than one myosuppressin receptor, and if so, whether they are differentially distributed in the premotor and motor neurons. One putative myosuppressin receptor had previously been identified from a *H. americanus* mixed tissue transcriptome (Christie et al. 2015). In the present study, transcriptomic analyses revealed four additional putative myosuppressin receptors in *H. americanus* neural tissues (MSR-II–V). Profiling of isolated premotor and motor neuron regions of the CG revealed expression of three of the MSRs (MSR-II, MSR-III, and MSR-IV) in the ganglion. Moreover, at least some of these receptors appear to be differentially expressed in the two neuron types, suggesting that differential receptor distribution may underlie, at least in part, the distinct physiological responses of the premotor and motor neurons to myosuppressin.

## MATERIALS AND METHODS

### Animals

Adult (~500 g) *H. americanus* were purchased from local seafood suppliers (Brunswick, ME); the individuals used included males and females and represented all stages of the molt cycle. Lobsters were housed in recirculating natural seawater aquaria and were maintained on a 12:12-h light-dark cycle at 10–12°C. The lobsters were fed a weekly diet of chopped shrimp or squid.

Individual lobsters were anesthetized by packing in ice for ~30 min before isolation of the heart from the cephalothoracic carapace via manual microdissection in chilled (8–10°C) physiological saline (composition in mM: 479.12 NaCl, 12.74 KCl, 13.67 CaCl_2_, 20.00 MgSO_4_, 3.91 Na_2_SO_4_, 11.45 Trizma base, and 4.82 maleic acid; pH 7.45; Dickinson et al. 2018). To isolate the CG, the heart was opened along the ventral axis, and the main trunk of the ganglion, along with lengths of the anterolateral nerves, was dissected from the surrounding musculature (Fig. 1*A*).

### Physiology

#### Separation of the premotor and motor neurons.

A single fiber taken from a length of 0.1-mm 6–0 suture silk was used as a ligature to tie a slack knot around the trunk of the CG just anterior to motor neuron 4 (Fig. 1*A*). Premotor neuron spike initiation zones extend from the most distal premotor neuron cell body to the soma of motor neuron 4 (Hartline 1967). This ligature placement ensured that the premotor neuron spike initiation zones were left intact, but were active only in the portion of the CG posterior to the ligature once it was tightened (Fig. 1*B*). Successful placement of the ligature was confirmed when only premotor neuron spikes were recorded on the trunk and only motor neuron spikes were recorded on the anterolateral nerves after the ligature was tightened. Because cutting the ganglion evokes injury discharges in recordings of both premotor and motor neurons in an intact ganglion, we cut the CG at the end of the experiment either just anterior or just posterior to the ligature; we then observed the bursting pattern of the cells on the nondisrupted side of the ligature to verify the ability of the ligature method to separate the cell types.

#### Recordings.

Petroleum jelly wells were built around small portions of the anterolateral nerves to monitor motor neuron output and around the trunk of the ganglion to monitor premotor neuron output (Fig. 1*A*; Williams et al. 2013). Bipolar stainless steel electrodes were used for extracellular recordings, with one electrode in the well and the other nearby in the bath. Neuronal output was amplified with a 1700 A-M Systems differential AC amplifier (Sequim, WA) and a 440 Brownlee Precision amplifier (Brownlee Instruments, San Jose, CA), digitized with a CED Micro 1401 digitizer, and recorded using Spike2 version 7.17 (Cambridge Electronic Design, Cambridge, UK), with a sampling rate of 10 kHz.

Temperature was maintained throughout recordings between 10°C and 12°C via an in-line Peltier temperature regulator (CL-100 bipolar temperature controller and SC-20 solution heater/cooler; Warner Instruments, Hamden, CT) with a temperature probe (Warner Instruments, Hamden, CT). Physiological saline was superfused at a flow rate of ~5 ml/min across the ganglion using a Rabbit peristaltic pump (Gilson, Middleton, WI). Myosuppressin (pQDLDHVFLRFamide), custom synthesized by GenScript Corporation (Piscataway, NJ), was introduced into the bath with the CG via the perfusion pump. Due to the relatively low aqueous solubility of myosuppressin, the peptide was dissolved in DMSO and then diluted in deionized water to make 10^−3^ M stock solutions containing 15% DMSO (Stevens et al. 2009). When diluted, the concentration of DMSO was at most 0.015%, which did not alter CG bursting patterns when superfused over the ganglion. Solutions were stored in small aliquots at −25°C and diluted in saline to the appropriate concentration directly preceding use.

After superperfusion of the intact CG with myosuppressin (10^−7^ or 10^−6^ M, 10-min peptide application) and a 45-min saline wash, the ligature was tightened to physically decouple the premotor and motor neurons, in an attempt to eliminate chemical and electrical communication between neuron types. After a return of bursting activity in isolated neuron types, the ligatured CG was again superfused with myosuppressin at 10^−7^ or 10^−6^ M.

#### Data analysis.

Physiological recordings were analyzed using functions built into Spike2 version 7.17 and scripts previously generated by the STG Laboratory at New Jersey Institute of Technology (NJIT)-Rutgers (https://centers.njit.edu/stglab/resources/). Data were averaged over 10 bursts, with control values taken shortly before the addition of the peptide to the bath and peptide values taken at the peak of the peptide effect, near the end of the 10-min peptide application. A burst was defined as a trail of at least five action potentials (“spikes”) occurring at a frequency of at least 100 Hz. Burst duration was quantified separately for the premotor and motor neurons in both intact and ligatured CG preparations as the duration from the first to the last spike in a given burst. Interburst interval was the length of time between successive bursts. Burst frequencies recorded in the premotor and motor neurons of the intact CG were identical, as premotor and motor neurons are coupled and burst in phase with one another (Williams et al. 2013). In ligatured CG preparations, the burst frequencies of the premotor and motor neurons were quantified separately, as the neurons had been physically decoupled. A trail of low-frequency tonic or irregular spikes (“leading spikes”) was recorded in 96% (25/26) of ligatured ganglia before the motor neuron bursts. These spikes did not reach the threshold for inclusion in the “burst” due to their lower frequency. These leading spikes were quantified separately as a characteristic of the ligatured motor neuron patterned output. Data were analyzed statistically and graphed using Prism version 7.0 (GraphPad Software, San Diego, CA). Of the 26 preparations in which the CG survived the tightening of the ligature, 18 were used for myosuppressin application and assessment of baseline burst characteristics of the ligatured ganglion, as well as for assessing the repatterning time of the ligatured neuron types. To standardize for variation in baseline, values are presented as percent change from baseline; only preparations that returned to baseline during the saline wash after peptide application were included in the analysis. The ROUT method for identifying outliers (Motulsky and Brown 2006) was applied using Prism. One-sample *t* tests were used to determine if the percentage change from baseline was significantly different from a hypothetical value of zero. Comparisons of two groups were done using Mann–Whitney tests. To compare more than two groups, ANOVAs were used, followed by Tukey post hoc tests. *P* values of <0.05 were considered significant; *n* values for all experiments refer to individual animals. All error values for physiological data represent standard deviation (SD).

### In Silico Identification of Putative H. americanus Myosuppressin Signaling Systems

#### Database searches.

Searches to identify transcripts encoding putative *H. americanus* myosuppressin precursors and receptor proteins were conducted with a workflow used previously for the identification of a variety of peptide precursors and receptors in this species, including those for myosuppressin (Christie et al. 2015, 2017). Specifically, the database of the online program tblastn (National Center for Biotechnology Information, Bethesda, MD; https://blast.ncbi.nlm.nih.gov/Blast.cgi) was set to Transcriptome Shotgun Assembly (TSA) and restricted to data from four lobster neural-specific transcriptomes: BioProject nos. PRJNA300643 [mixed nervous system regions; brain, abdominal nerve cord, stomatogastric nervous system (STNS), and CG; Northcutt et al. 2016)], PRJNA338672 (eyestalk ganglia specific; Christie et al. 2017), PRJNA379629 (brain specific; Christie et al. 2018a), and PRJNA412549 (CG specific; Christie et al. 2018b). In searches for transcripts encoding putative myosuppressin precursors (which were limited to the CG-specific transcriptome), a previously identified *H. americanus* myosuppressin preprohormone (accession no. ACX46385; Stevens et al. 2009) was used as the query protein. In searches for putative *H. americanus* myosuppressin receptor-encoding transcripts, a previously predicted *Homarus* receptor (renamed here MSR-I, deduced from accession no. GEBG01049137; Christie et al. 2015) was used as the query sequence.

#### Identification of myosuppressin peptide and precursors.

The putative mature structures of the *H. americanus* CG myosuppressin and myosuppressin precursor-related peptides were predicted using a workflow employed previously for peptide discovery in this species, including myosuppressin in other portions of the nervous system (Christie et al. 2015, 2017). In brief, BLAST hits were translated using the Translate tool of ExPASy (https://web.expasy.org/translate/) and assessed for the presence of a signal peptide using the online program SignalP 3.0 (https://www.cbs.dtu.dk/services/SignalP/; Bendtsen et al. 2004). Prohormone cleavage sites were identified based on homology to known myosuppressin preprohormone processing schemes (Christie et al. 2015, 2017). The sulfation state of tyrosine residues and disulfide bonding between cysteine residues were predicted using the online programs Sulfinator (https://web.expasy.org/sulfinator/; Monigatti et al. 2002) and DiANNA (http://clavius.bc.edu/~clotelab/DiANNA/; Ferré and Clote 2005). Cyclization of NH_2_-terminal glutamine residues and COOH-terminal amidation at glycine residues were predicted by homology to mass-spectrally identified decapod myosuppressin isoforms (Stemmler et al. 2007).

#### Identification and vetting of candidate receptors.

Candidate myosuppressin receptors were predicted and vetted using a workflow that previously identified putative peptide receptors in a variety of decapod species, including *H. americanus* (Christie et al. 2015; Christie and Yu 2019; Dickinson et al. 2019b). Specifically, nucleotide sequences were translated using the Translate tool of ExPASy (https://web.expasy.org/translate/) and assessed for completeness. Next, each *H. americanus* receptor was used as the input query in a BLAST search of the annotated *Drosophila melanogaster* proteins curated in FlyBase (version FB2019_06; http://flybase.org/blast/index.html; Thurmond et al. 2019). This workflow was conducted on or before January 14, 2020. Finally, protein structural motifs were predicted for each of the *H. americanus* receptors using the online program Pfam (version 32.0; http://pfam.xfam.org; El-Gebali et al. 2019). Transmembrane domains were predicted using the TOPCONS web server (Tsirigos et al. 2015).

#### Amino acid alignments and calculations of amino acid identity/similarity.

Amino acid alignments were done using the online program MAFFT (version 7; https://mafft.cbrc.jp/alignment/software/; Katoh and Standley 2013). Amino acid identity/similarity between putative peptide receptors was calculated using the MAFFT alignment outputs. Specifically, percent identity was calculated as the number of identical amino acids divided by the total number of residues in the longest sequence (multiplied by 100), while amino acid similarity was calculated as the number of identical and similar amino acids divided by the total number of residues in the longest sequence (multiplied by 100).

#### Phylogenetic analysis of Homarus myosuppressin receptors.

Phylogenetic relationships between the putative *H. americanus* myosuppressin receptor sequences and defined *D. melanogaster* peptide receptors were inferred from a multiple sequence alignment constructed using default MUSCLE (Edgar 2004) settings in Geneious (version 10.1.3; Biomatters Ltd., Auckland, New Zealand; Kearse et al. 2012). Evolutionary analyses were conducted in MEGA X (Kumar et al. 2018) using the maximum likelihood method based on the Le and Gascuel (2008) model. Initial tree(s) for the heuristic search were obtained automatically by applying Neighbor-Joining and BioNJ algorithms to a matrix of pairwise distances estimated using the Jones–Taylor–Thornton model (Jones et al. 1992) and then selecting the topology with superior log likelihood value. A discrete gamma distribution was used to model evolutionary rate differences among sites [5 categories (+*G*, parameter = 1.2975)]. The resulting tree was drawn to scale with bootstrap support from 1,000 iterations indicated at branch nodes and branch lengths measured in terms of substitutions per site. The analysis involved 40 amino acid sequences. All positions with less than 95% site coverage were eliminated such that fewer than 5% alignment gaps, missing data, and ambiguous bases were allowed at any position; the final data set consisted of 287 positions. Phylogenetic inferences made using neighbor joining (Saitou and Nei 1987) and minimum evolution (Rzhetsky and Nei 1992) approaches generated trees with similar topologies. Accession numbers for sequences used in the phylogenetic analyses are provided in Supplemental Table S1 (all Supplemental Tables are available at https://zenodo.org/record/3678732#.XlBhjChKhPY).

### Reverse Transcriptase PCR

#### Myosuppressin receptor cloning.

To facilitate cloning of the putative *H. americanus* myosuppressin receptors, total RNAs were purified from individual brains and eyestalk ganglia pairs (*n* = 3 independent samples from each tissue) as described previously (Christie et al. 2017, 2018a). RNA quality and quantity were assessed using an Agilent 2100 Bioanalyzer (Agilent Technologies, Santa Clara, CA). cDNAs were synthesized from ~500 ng of total RNA with random pentadecamers (IDT, San Diego, CA) and a SuperScript III First-Strand synthesis system (Life Technologies Corp.). Full-length transcripts corresponding to MSR-I, MSR-II, and MSR-V were amplified using the respective cDNAs with SapphireAmp Fast PCR master mix (Takara Bio USA, Inc., Mountain View, CA) and oligonucleotide primers (Table 1) designed to span the respective open reading frames (ORFs). PCR was performed in a 20-μL reaction volume with 0.5-μL of cDNA and thermocycler conditions consisting of 95°C for 2 min, followed by 40 cycles of 95°C for 20 s, 56°C for 20 s, and 72°C for 2 min, with a final extension at 72°C for 5 min. Overlap extension PCR (Wurch et al. 1998) was used to amplify the MSR-IV ORF with oligonucleotide primers designed to stagger the MSR-IV coding sequence (Table 1). PCR was performed as before with SapphireAmp Fast PCR master mix in a 20-μL reaction volume with 0.5-μL of cDNA and initial thermocycler conditions consisting of 95°C for 2 min, followed by 37 cycles of 95°C for 20 s, 56°C for 20 s, and 72°C for 1 min, with a final extension at 72°C for 5 min. The respective products were then used as templates for a second round of PCR using primers designed to span the putative ORF and thermocycler conditions consisting of 95°C for 2 min, followed by 27 cycles of 95°C for 20 s, 56°C for 20 s, and 72°C for 1:25 min, with a final extension at 72°C for 5 min. The *H. americanus* MSR-III sequence predicted in the transcriptomic assembly is a 3′ fragment that lacks an identifiable start codon. Consequently, primers (Table 1) were designed to amplify a 931-bp portion of the fragment that included the putative stop codon. PCR was performed as before with 0.5-μL of cDNA and thermocycler conditions consisting of 95°C for 2 min, followed by 40 cycles of 95°C for 20 s, 58°C for 20 s, and 72°C for 1 min, with a final extension at 72°C for 5 min. All PCR products were visualized on 1.5% agarose gels stained with SYBR Safe (Life Technologies Corp.), subcloned into pCR2.1TOPO TA (Life Technologies Corp.), and sequenced at the Arizona State University DNA core laboratory (Tempe, AZ). Consensus sequences for the cloned MSR transcripts have been deposited in GenBank under accession nos. MT068477–MT068483.

**Table 1.** Oligonucleotide primers used

Primer	Sequence (5′ → 3′)
*Cloning/expression profiling*	
HaMS start F	ATGGTGTTCCGCAGCTG
HaMS stop R	TTATTGCTGGGATCGTCCGA
*Cloning*	
HaMSR-I start F	ATGGAGCAGGTGGAGGC
HaMSR-I stop R	TCAGACATGTGTGATACATGTG
HaMSR-II start F	ATGTTTAGTGTTAACTTTAGCGAG
HaMSR-II stop R	TCAGACATGTGTGATGCAG
HaMSR-III 350	CCGTGATCTGCAACATCC
HaMSR-III stop R	TCACCAAACTCTGGTGTGTTCC
HaMSR-IV start F	ATGATGACTGCGGGGAGC
HaMSR-IV 755 R	TGTAGCAGGCCATTCGAT
HaMSR-IV 252 F	CTTGGCGCTGATGATCTG
HaMSR-IV stop R	TTAGAGCTGGGTAGAAACTGTC
HaMSR-V start F	ATGGAGCGGTCCCTGC
HaMSR-V stop R	TCATATCTTAGTGTTAAGAACTTTGC
*Expression profiling*	
HaMSR-I 653 F	ACTTCACCATCAGCACGA
HaMSR-I 1173 R	GCGGATAGATAGCACCGA
HaMSR-II 179 F	CCACCACACAAGACTCCT
HaMSR-II 682 R	GGATGTTGCAGATGACGG
HaMSR-III 350 F	CCGTGATCTGCAACATCC
HaMSR-III 816 R	CAGCAGGAAGTTGATGGC
HaMSR-IV 252 F	CTTGGCGCTGATGATCTG
HaMSR-IV 755 R	TGTAGCAGGCCATTCGAT
HaMSR-V 695 F	TGTCCAACGATGACGGAT
HaMSR-V 1229 R	TTGATGAGCGCCAACAAG
HaGAPDH 96 F	TCGGTCGTCTTGTCCTTC
HaGAPDH 599 R	CAGTGACGGCATGAACAG

F, forward; GAPDH, glyceraldehyde-3-phosphate dehydrogenase; Ha, *Homarus americanus*; MS, myosuppressin; MSR, myosuppressin receptor; R, reverse.

#### Myosuppressin cloning.

To clone the *H. americanus* myosuppressin preprohormone coding sequence, total RNAs were purified from isolated complete CGs as described previously (Christie et al. 2018b) and then treated with DNase I (New England Biolabs, Ipswich, MA) for 10 min at 37°C to remove contaminating genomic DNA. cDNAs were synthesized using a SuperScript III First-Strand synthesis system (Life Technologies) from ~100 ng of total RNA with random pentadecamers (IDT). The myosuppressin preprohormone was amplified with SapphireAmp Fast PCR master mix (Takara Bio USA, Inc.) and oligonucleotide primers (Table 1) designed to the ORF in the deposited *H. americanus* myosuppressin preprohormone mRNA sequence (accession no. GQ303179). PCR was performed in a 20-μL reaction volume with 0.5 μL of cDNA and thermocycler conditions consisting of 95°C for 2 min, followed by 40 cycles of 95°C for 20 s, 56°C for 20 s, and 72°C for 30 s, with a final extension at 72°C for 5 min. The PCR product was visualized, subcloned, and sequenced as above.

#### Transcript expression profiling.

To examine expression of the myosuppressin and MSR transcripts, total RNAs were purified from the premotor and motor neuron regions of the CG. Each CG was cut just posteriorly of motor neuron 5 to separate all premotor and motor neuron cell bodies. For each sample, tissue from 10 ganglia was pooled (*n* = 6 pooled biological replicates for premotor neurons; *n* = 7 for motor neurons). RNA purification was performed using a Takara NucleoSpin XS RNA isolation kit (Takara Bio USA, Inc.) with on-column rDNase treatments based on manufacturer protocols. Prior to storage at −80°C, RNA quantity and quality were assessed with an Agilent 2100 Bioanalyzer (Agilent Technologies, Santa Clara, CA) or an Agilent 4150 TapeStation system (Agilent Technologies) with RNA ScreenTape analysis. cDNAs were synthesized from premotor and motor neuron tissue RNA (2–82 ng of total RNA) as above with random pentadecamers. The myosuppressin preprohormone transcript was amplified using primers that spanned the full ORF, whereas the respective MSRs were amplified using primers designed to amplify 500-bp fragments of each transcript (Table 1). Amplification was performed with SapphireAmp Fast PCR master mix (Takara Bio USA, Inc) in a 20-μL reaction volume with 0.8 μL of cDNA and thermocycler conditions consisting of 95°C for 2 min, followed by 40 cycles of 95°C for 20 s, 56°C for 20 s, and 72°C for 30 s, with a final extension at 72°C for 5 min. The PCR product was visualized, subcloned, and sequenced as above. To confirm the suitability of the respective primer sets for amplification, aliquots of brain and eyestalk ganglia, described above, were also profiled using the same conditions as the premotor and motor neuron regions. To verify the integrity of the cDNA templates, a 500-bp fragment of the *H. americanus* glyceraldehyde-3-phosphate dehydrogenase (GAPDH) housekeeping gene (accession no. FE043664) was likewise amplified. PCR products were separated on 1.5% agarose gels and visualized as before with representative amplicons subcloned and sequence verified. Gel images were obtained using an Azure 200 gel imaging workstation (Azure Biosystems, Dublin, CA) and then processed in Photoshop CS6 v13.0 (Adobe Systems Inc., San Jose, CA).

## RESULTS

**Fig. 2.**
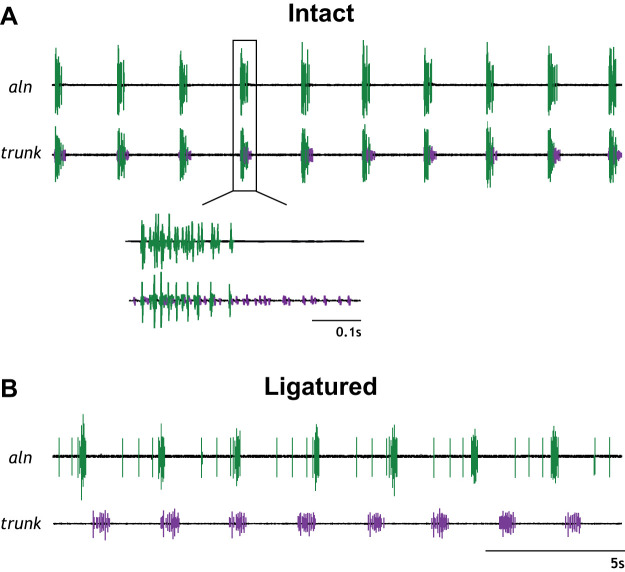
Baseline firing pattern differs in the intact and ligatured cardiac ganglion (CG). *A*: in the intact CG, the premotor (purple) and motor (green) neuron bursts are in-phase, with the premotor bursts beginning just before motor neuron bursting. In most preparations (19/25), only motor neuron bursting was captured by the well on the anterolateral nerve (aln), while the well on the trunk captured both premotor and motor bursting activity. *Inset*: a single burst at a higher recording speed shows that the burst of small premotor neuron spikes (purple) starts before and ends after the burst of larger motor neuron spikes (green). *B*: when the CG was ligatured, the premotor and motor bursts exhibited bursting patterns independently of one another. Baseline firing frequency of the motor neurons was slower, with longer baseline burst durations in the premotor firing pattern.

After the CG had been removed from the surrounding musculature, the isolated ganglion always displayed regular bursting activity. Control activity of the isolated CG consisted of spontaneous, coupled bursting activity. Motor neuron output was monitored distally of motor neurons 1 and 2 on the anterolateral nerves. Although Hartline (1967) reported that premotor axons do not extend distally from these motor cell bodies, in 19% (5/26) of recordings, premotor neuron spikes were recorded from this region of the ganglion. The well on the posterior trunk consistently captured both premotor and motor neuron activity in the intact CG, where axons for both neuronal types overlap in the ganglionic trunk (Hartline 1967). Sorting the recorded action potentials by size allowed for analysis of the neuronal firing pattern. In the intact CG, premotor and motor neuron activity was in-phase (Fig. 2*A*). Premotor bursts began milliseconds before and always ended after the motor neuron bursts, but the bursts ended at variable phases, as previously described (Cooke 2002; Mayeri 1973; Williams et al. 2013). Thus premotor bursts were longer than the coupled motor bursts (Fig. 2*A*).

### The Ligature is an Effective Method to Separate the Premotor and Motor Neurons

To understand the different neuronal components of the CG, the ligature placed before experimentation was tightened to physically decouple the premotor and motor neurons. Separation of cell types by ligature was an effective method to physically decouple premotor and motor neurons. Tying a small knot from a single strand of 6-0 suture silk did not obstruct superfusion and caused minimal damage to the cell bodies and axons. Placement of the ligature anterior to the fourth motor neuron allowed the spike initiation zones of the premotor axons to remain intact while the neuronal types were decoupled (Fig. 1*B*). To ensure that the ligature was an effective method of separating the neuronal types, we cut the ganglion after tightening the ligature in several preparations. When the ganglionic trunk was cut (data not shown) just posterior to the ligature, motor neuron bursting continued without an injury discharge (*n* = 3). When the cut was made just anterior to the ligature, premotor neuron bursting was undisrupted (*n* = 3), indicating that the ligature effectively separated the two regions of the CG.

### The Ligatured Cardiac Ganglion Reestablishes Bursting Activity in Both the Premotor and Motor Neurons

Following tightening of the ligature, the coordinated bursting activity characteristic of the intact CG was abolished, replaced by uncoupled, spontaneous neuronal output (Fig. 2*B*). The pin electrode on the anterolateral nerve recorded only motor neuron output, and the pin electrode on the trunk recorded only premotor neuron activity. Of 41 ligatured experiments attempted, both the premotor and motor neurons reestablished an observable bursting pattern in 26 CGs (63.4%). However, the baseline firing pattern of both the premotor and motor neurons changed relative to their intact firing pattern (Fig. 2).

**Fig. 3.**
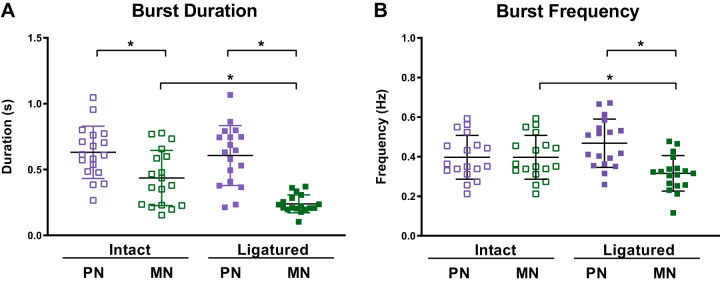
Baseline burst characteristics of the intact and ligatured cardiac ganglion (CG). *A*: burst duration of the premotor neurons (PN) did not change with the ligature (0.63 ± 0.19 s vs. 0.6 ± 0.2 s), but tightening elicited a significant decrease in burst duration of the motor neurons (MN; 0.4 ± 0.2 s vs. 0.24 ± 0.07 s). *B*: frequency of bursts in the premotor neurons did not change with the ligature (0.40 ± 0.11 Hz vs. 0.47 ± 0.12 Hz), but ligaturing the CG elicited a significant decrease in motor neuron burst frequency (0.32 ± 0.09 Hz vs. 0.40 ± 0.11 Hz). Error bars indicate SD. *P < 0.05, significant change with tightening of the ligature (1-way ANOVAs with post hoc Tukey tests; burst duration: intact PN vs. MN, *P* < 0.0001; intact vs. ligatured MN, *P* = 0.0042; ligatured PN vs. MN, *P* < 0.0001; *n* = 18 preparations; burst frequency: intact vs. ligatured MN, *P* = 0.0338; ligatured PN vs. MN, *P* = 0.0005, *n* = 18 preparations).

In the 18 preparations in which myosuppressin was applied to the intact and ligatured CG, changes in baseline burst duration and frequency induced by ligature tightening were examined across both neuron types (Fig. 3). The duration of bursts in the ligatured motor neurons was shorter than that of bursts in the motor neurons in the intact CG (0.24 ± 0.07 vs. 0.4 ± 0.2 s; *P* = 0.0042). In contrast, the burst duration of the premotor neurons did not change with the ligature (0.63 ± 0.19 vs. 0.6 ± 0.2 s; *P* = 0.9757). The burst frequency of ligatured motor neurons was significantly lower than that of the motor neurons in the intact CG (0.32 ± 0.09 vs. 0.40 ± 0.11 Hz; *P* = 0.0338), but the frequency of bursting in the premotor neurons did not change with the tightening of the ligature (0.40 ± 0.11 vs. 0.47 ± 0.12 Hz; *P* = 0.1123).

In the ligatured CG, the duration of premotor neuron bursts was longer than that of the motor neuron bursts (0.6 ± 0.2 vs. 0.24 ± 0.07 s; *P* < 0.0001). In contrast, burst frequency, which was identical in the two neuronal types in the intact ganglion, was higher in the ligatured premotor neurons than in the ligatured motor neurons (0.47 ± 0.12 vs. 0.32 ± 0.09 Hz; *P* = 0.0005). Additionally, leading spikes were recorded in 96% (25/26) of ligatured ganglia before the motor neuron bursts. Similar leading spikes were recorded in 19% (5/26) of ligatured premotor neurons (Fig. 2*B*).

Across all 18 preparations in which bursting was recorded as the ligature was tightened and during the reestablishment of bursting in the two neuronal types, there was considerable variability in the time required to reestablish bursting in the two neuron types (means: motor, 4.33 ± 8.48 min; premotor, 10.47 ± 13.51 min; paired *t* test, *P* = 0.1346, *n* = 18 preparations). In 13 of the 18 preparations, the bursting pattern was reestablished more quickly in the motor neurons than in the premotor neurons (binominal test, *P* = 0.049). In three preparations, the postligature pattern was established immediately in both cell types. In the remaining two preparations, postligature bursting was established more quickly in the premotor neurons. In 36.5% (15/41) of preparations, a strong postligature burst pattern was never achieved; of these 15 CGs, 4 (9.8%) demonstrated no bursting in either cell type after the ligature. Of the 11 ganglia in which only one neuron type reestablished a bursting pattern, motor neurons achieved a postligature burst pattern in 5 CGs, while the premotor neurons reestablished bursting in the other 6 CGs.

### Myosuppressin Modulates Cardiac Ganglion Output

**Fig. 4.**
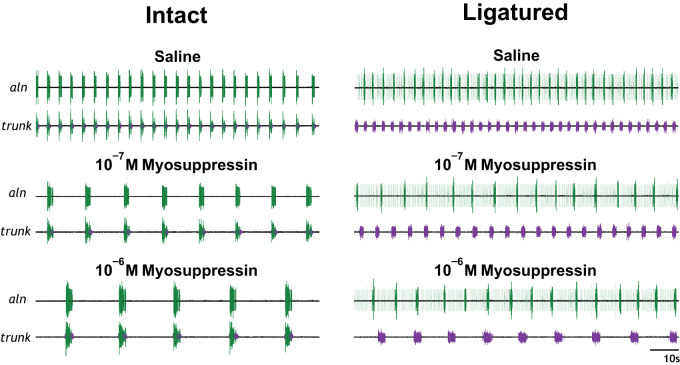
Bursting activity of the intact and ligatured cardiac ganglion (CG) in response to myosuppressin application. At both 10^−7^ M and 10^−6^ M, myosuppressin application qualitatively altered the bursting pattern of the intact and ligatured premotor (purple) and motor (green) neurons. In the ligatured CG, myosuppressin differentially altered the bursting pattern of the premotor and motor neurons. All extracellular traces are from the same individual. aln, Anterolateral nerve.

**Fig. 5.**
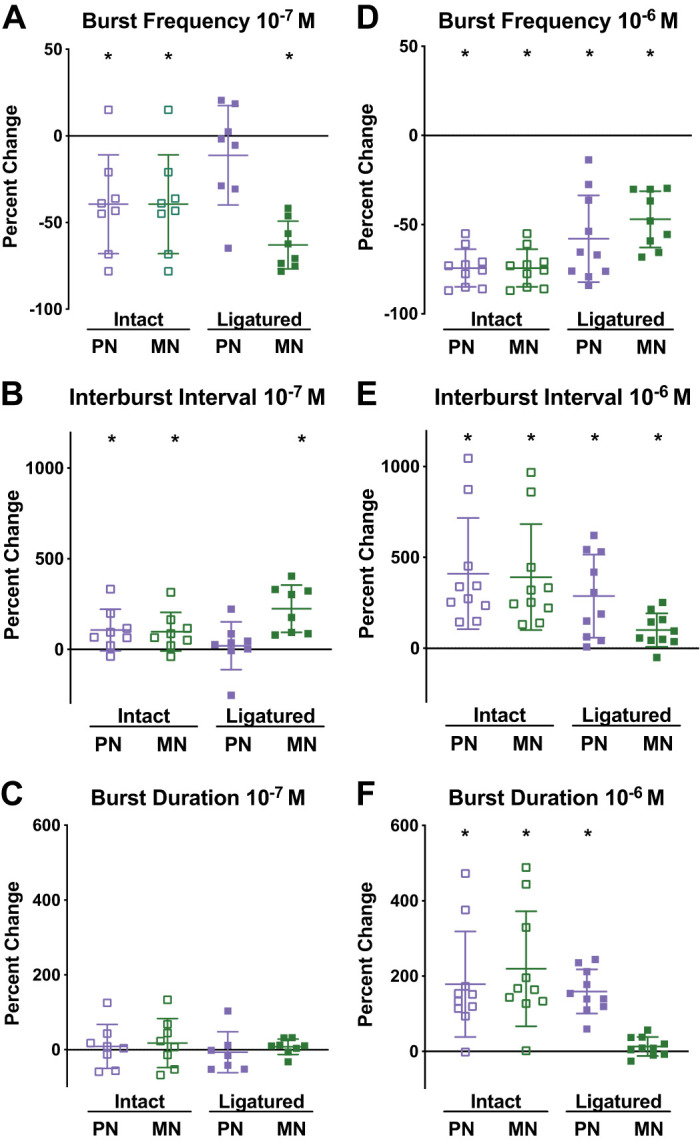
Myosuppressin altered the burst characteristics of the intact and ligatured ganglion. Myosuppressin elicited changes in burst frequency (*A* and *D*), interburst interval (*B* and *E*), and burst duration (*C* and *F*) at 10^−7^ M (*A–C*; *n* = 8 preparations) and 10^−6^ M (D*–*F; *n* = 10 preparations) in the intact and ligatured CG. The ROUT method was used to eliminate outliers. Error bars indicate SD. *P < 0.05, significant change from baseline (1-sample *t* tests; burst frequency, 10^−7^ M: PN and MN, intact, *P* = 0.0058; MN ligatured, *P* < 0.0001; interburst interval, 10^−7^ M: PN intact, *P* = 0.0332; MN intact, *P* = 0.0365; MN ligatured, *P* = 0.0018; burst frequency, 10^−6^ M: PN and MN, intact and ligatured, *P* < 0.0001; burst duration, 10^−6^ M: PN intact, *P* = 0.0030; MN intact, *P* = 0.0014; PN ligatured, *P* < 0.0001; interburst interval, 10^−6^ M: PN intact, *P* = 0.0022; MN intact, *P* = 0.0021; PN ligatured, *P* = 0.0033; MN ligatured, *P* = 0.0075). Changes in both neuronal types were larger in magnitude in response to 10^−6^ M myosuppressin than in response to 10^−7^ M myosuppressin (Mann–Whitney tests; burst frequency: PN and MN, *P* = 0.0044; burst duration: PN, *P* = 0.0031; MN, *P* < 0.0001; interburst interval: PN and MN, *P* = 0.0021).

Application of myosuppressin to the isolated but intact CG resulted in observable changes in burst characteristics (Fig. 4), consistent with previous reports (Stevens et al. 2009). To quantify the effects of myosuppressin, burst characteristics, including burst frequency, interburst interval, and burst duration, were measured at the time of peak response to the peptide. To enable comparison between CGs with different baseline firing patterns, data were normalized by comparing percent change from baseline (Fig. 5, *A*–*F*). When superfused over the intact CG at a concentration of 10^−7^ M, myosuppressin elicited a decrease in burst frequency (Fig. 5*A*; *P* = 0.0058 for both cell types) and an increase in interburst interval (Fig. 5*B*; *P* = 0.0332 for premotor neurons, *P* = 0.0365 for motor neurons). No change in burst duration was observed for either motor or premotor neurons (Fig. 5*C*). When superfused at 10^−6^ M, myosuppressin also elicited a decrease in burst frequency (Fig. 5*D*; *P* < 0.0001 for both cell types) and an increase in interburst interval (Fig. 5*E*; *P* = 0.0022 for premotor neurons, *P* = 0.0021 for motor neurons) in both cell types. Additionally, the peptide elicited a large increase in burst duration in both neuronal types (Fig. 5*F*; *P* = 0.0030 for premotor neurons, *P* = 0.0014 for motor neurons). All observed changes in burst characteristics resulting from 10^−6^ M peptide application were larger in magnitude than those elicited in response to 10^−7^ M application (*P* < 0.05).

Application of myosuppressin to the ligatured CG enabled us to determine whether the peptide exerted independent modulatory effects on the premotor and motor neurons of the CG. Myosuppressin superfused at both 10^−7^ M and 10^−6^ M altered specific aspects of the bursting pattern in both the premotor and motor neurons (Fig. 4). Although 10^−7^ M myosuppressin elicited a decrease in burst frequency in both neuronal types when the CG was intact, in the ligatured CG, burst frequency was decreased only in the motor neurons (Fig. 5*A*; *P* < 0.0001 for motor neurons, *n* = 8); 10^−7^ M myosuppressin did not significantly change burst frequency in the premotor neurons (Fig. 5*A*; *P* = 0.3045, *n* = 8). When myosuppressin was superfused over the ligatured CG at a higher concentration (10^−6^ M), a decrease in burst frequency during myosuppressin application was observed in both neuron types (Fig. 5*D*; *P* < 0.0001 for both premotor and motor neurons, *n* = 10).

When superfused at 10^−7^ M, myosuppressin elicited a significant increase in interburst interval only in the ligatured motor neurons (Fig. 5*B*; *P* = 0.0018), while an increase had been observed in both cell types in the intact CG. However, an increase in interburst interval was observed in both cell types at a concentration of 10^−6^ M (Fig. 5*E*; *P* = 0.0033 for premotor neurons, *P* = 0.0075 for motor neurons), as in the intact ganglion.

As was the case in the intact ganglion, 10^−7^ M myosuppressin did not alter burst duration in either the ligatured premotor or motor neurons, although an increase in burst duration was observed in the ligatured premotor neurons in the presence of 10^−6^ M myosuppressin (Fig. 5, *C* and *F*; *P* < 0.0001 for premotor neurons). However, 10^−6^ M myosuppressin failed to elicit a change in burst duration in the motor neurons, which contrasts with its effects in the intact ganglion, where it elicited an increase in burst duration in both premotor and motor neurons.

**Fig. 6.**
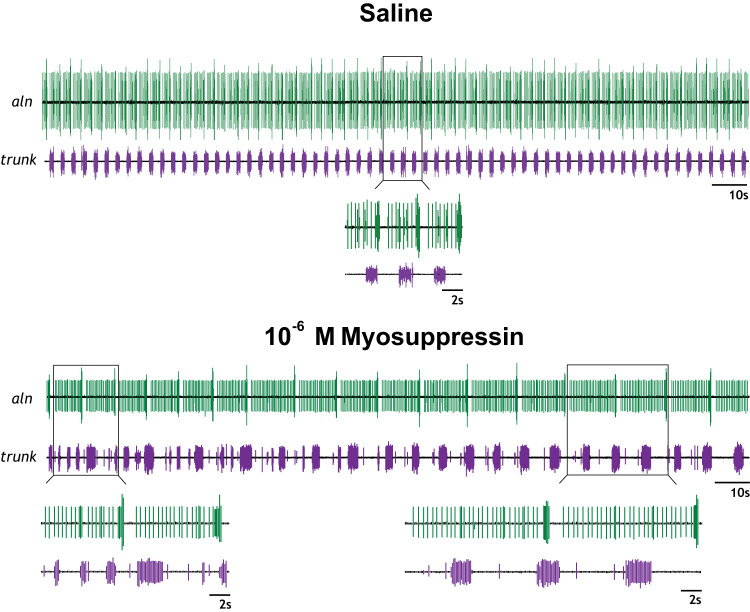
Response to myosuppressin in the ligatured premotor neurons has a later onset than in the motor neurons. Bursting activity of the premotor (purple) and motor (green) neurons is shown in a ligatured cardiac ganglion (CG) preparation before myosuppressin application (saline) and during the final 200 s of the 10-min 10^−6^ M myosuppressin application. Burst frequency of the ligatured motor neurons decreased continuously during peptide application. Large-magnitude increases in burst duration and decreases in burst frequency in the premotor neurons appeared later during peptide application, as the shorter bursts of the ligatured premotor neurons were overtaken by a pattern of longer bursts at lower frequency. aln, Anterolateral nerve.

While changes in various burst characteristics were observed across the cell types at the peak response to the peptide, the time course of these alterations to the bursting pattern differed between the two neuronal types (Fig. 6). At a concentration of 10^−6^ M, the onset of the characteristic decrease in burst frequency observed in the motor neurons was gradual across the period of peptide application. The onset of this gradual decrease consistently preceded the large increase in burst duration observed in the premotor cells. The number of leading spikes that preceded ligatured motor neuron bursts was not significantly altered by myosuppressin application at 10^−6^ M or 10^−7^ M.

Taken together, these data suggest that myosuppressin exerts distinct and concentration-dependent effects on the premotor and motor neurons of the CG. These data raise two additional questions. First, having noted that the peptide concentrations that elicit these effects are concentrations typically associated with local rather than hormonal release (Dickinson et al. 2015), we asked whether myosuppressin might be produced locally in the CG. Second, given the different responses to myosuppressin in the two neuronal types and the fact that multiple myosuppressin receptors have been identified in at least some arthropod species (Dickinson et al. 2019b; Egerod et al. 2003), we asked whether multiple myosuppressin receptors are expressed in the lobster nervous system, and if so, whether they are differentially distributed across the two neuronal types.

### In Silico Identification of Myosuppressin as a Neuropeptide Produced in the Cardiac Ganglion of H. americanus

The threshold concentrations for the myosuppressin effects reported here were 10^−7^ or 10^−6^ M, concentrations typically associated with locally released peptide. While several peptides have been identified previously in the *H. americanus* CG (Christie et al. 2010b; Dickinson et al. 2015, 2018), including at least one, diuretic hormone 31, synthesized by the motor neurons (Christie et al. 2010b), the presence of myosuppressin within the ganglion has not been investigated. Here, using a previously identified *Homarus* prepromyosuppressin sequence (Stevens et al. 2009), the *H. americanus* CG-specific assembly was searched for transcripts encoding putative homologs. This search returned three transcripts (accession nos. GGPK01064738 – GGPK01064740) that encode the same 100-amino acid full-length preprohormone. The putative CG prepromyosuppressin is identical to that identified initially by Stevens et al. (2009) and is predicted to give rise to four peptides: the myosuppressin isoform pQDLDHVFLRFamide and the linker/precursor-related peptides VCVGVGETMPPPICLSQQVPLSPFA (disulfide bridging between the two cysteine residues), LCSALINISEFSRAMEEY_(SO__3__H)_LGAQAIERSMPVNEPEV, and SQQ. Furthermore, multiple clones with >99% nucleotide (nt) identity to the in silico-derived transcripts were amplified from CG-specific cDNAs.

### Identification of Five Candidate H. americanus Neuronal Myosuppressin Receptors

**Fig. 7.**
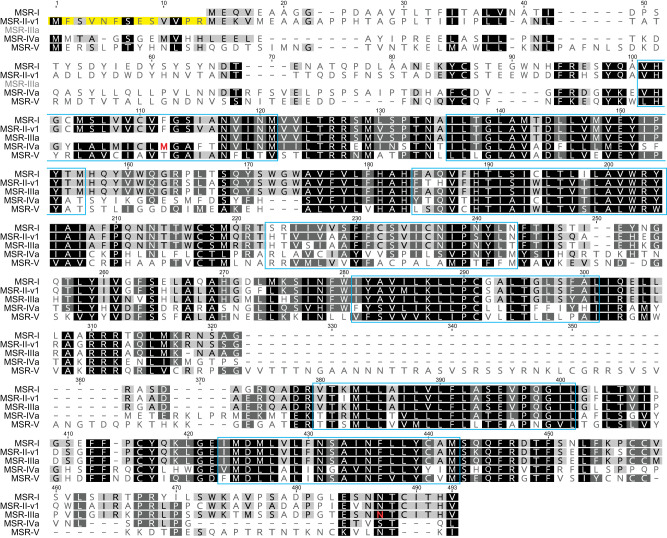
MAFFT alignment of select putative *Homarus americanus* myosuppressin receptors (MSRs). All proteins identified and shown are full-length sequences except for MSR-IIIa, which is a carboxyl-terminal partial protein. Relative positions of predicted transmembrane domains are indicated by blue boxes. The 13-amino acid amino-terminal extension that distinguishes MSR-II-v1 from MSR-II-v2 (not shown) is highlighted in yellow. The single amino acid substitutions that distinguish MSR-III “a” and “b” morphs (asparagine in IIIa vs. threonine in IIIb; not shown) and MSR-IV “a” and “b” morphs (methionine in IVa vs. valine in IVb; not shown) are colored red. MSR-I, myosuppressin receptor I; MSR-II-v1, myosuppressin receptor II variant 1; MSR-IIIa, myosuppressin receptor IIIa; MSR-IVa, myosuppressin receptor IVa; MSR-V, myosuppressin receptor V.

Prior to the study presented here, a single putative myosuppressin receptor had been reported from *H. americanus* (Christie et al. 2015). This receptor (renamed here MSR-I; Fig. 7) was predicted from a transcript identified via a BLAST search of a *H. americanus* mixed nervous system region transcriptome using a *D. melanogaster* MSR as the input query (Christie et al. 2015). Because a BLAST search of the *Homarus* CG-specific assembly failed to identify any transcripts encoding this protein (Table 2 and Supplemental Table S1), we hypothesized that additional receptors for myosuppressin must be present in *H. americanus*, including in the CG. Reassessment of the mixed nervous system assembly, as well as searches of brain-, eyestalk ganglia-, and CG-specific transcriptomes, identified sequences encoding four additional candidate *H. americanus* MSRs (MSR-II–V; Fig. 6 and Supplemental Table S2).

**Table 2.** In silico detection of putative myosuppressin receptors in the nervous system of Homarus americanus

	Receptor				
Assembly	MSR-I	MSR-II	MSR-III	MSR-IV	MSR-V
Mixed	+	+	+	+	+
Br	+	+	+*	+	+
EG	+	+	+	+	+
CG	−	+†	+	+	−

Plus and minus signs indicate detection and nondetection, respectively, of mixed nervous system (Mixed), brain-specific (Br), eyestalk ganglia-specific (EG), and cardiac ganglion-specific (CG) assembly in myosuppressin receptors (MSR). Receptors (with reference for first identification) are MSR-I (Christie et al. 2015), MSR-II (this study), MSR-III (this study), MSR-IV (this study), and MSR-V (this study).

* A 131-amino acid internal fragment that differs from the corresponding portion of MSR-III at 5 positions (3 conservative and 2 nonconservative substitutions) was predicted from the brain-specific assembly. Whether this partial protein represents a variant of MSR-III or an additional myosuppressin receptor (MSR-VI) remains to be determined.

† Two splice variants of MSR-II appear to be expressed in the CG.

**Fig. 8.**
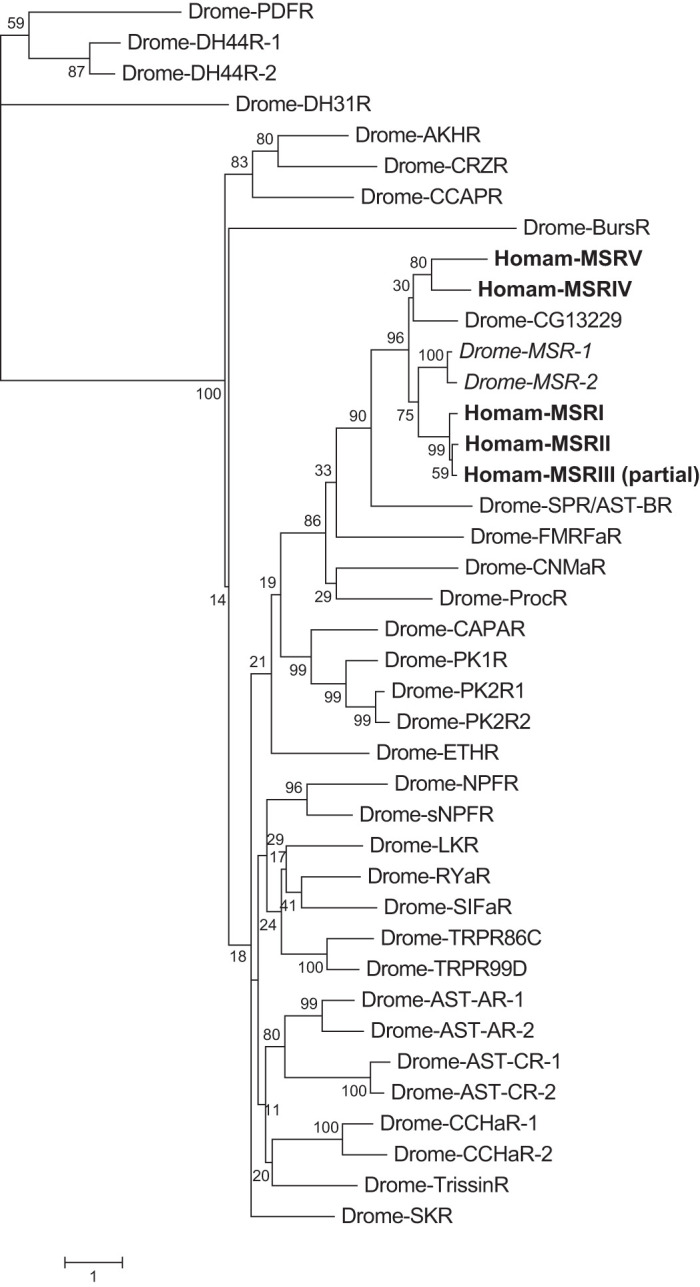
Maximum likelihood tree depicting phylogenetic relationships among putative *Homarus americanus* myosuppressin receptors and *Drosophila melanogaster* peptide receptors. The tree with the highest log likelihood is shown, and the percentage of trees in which the associated taxa clustered together (1,000 replicates) is shown next to the branches. The tree is drawn to scale, with branch lengths measured in the number of substitutions per site. Drome MSRs functionally characterized as myosuppressin receptors (Egerod et al. 2003; Johnson et al. 2003) are indicated in italics.

Unlike *Homarus* MSR-I, whose top FlyBase annotated *D. melanogaster* protein hit is isoform B of myosuppressin receptor 1 (accession no. AGB94019), MSR-II–V returned uncharacterized protein Dmel_CG13229 (accession no. AGB94019) as the most similar protein. Although it was originally identified as a putative *D. melanogaster* myosuppressin receptor, CG13229 was not activated by the native *Drosophila* myosuppressin isoform, at least in the expression system/bioassay that was used for receptor deorphanization (Hauser et al. 2006). Regardless, all four of the new candidate *H. americanus* MSRs are similar in amino acid sequence to MSR-I (Table 3) and possess a single serpentine receptor class W seven-transmembrane domain (Fig. 7), a domain also present in *Homarus* MSR-I and *D. melanogaster* myosuppressin receptor 1 and CG13229. Furthermore, the putative *Homarus* MSRs cluster in an MSR-specific clade with the *Drosophila* proteins in which the CG13229 sequence forms a well-supported branch with the *Homarus* MSR-IV and MSR-V proteins (Fig. 8). While transcripts encoding *H. americanus* MSR-I–V were found in the mixed nervous system, brain-specific, and eyestalk ganglia-specific transcriptomes, evidence of expression for only MSR-II–IV was found in the CG-specific assembly (Table 2 and Supplemental Table S1). Clones amplified from brain and eyestalk ganglia cDNAs for MSR-I and -MSR-II exhibited >99% nt identity with the transcriptomic sequences, as did partial clones comprising a 931-bp portion of the MSR-III 3′ fragment. Three MSR-IV variants (referred to as MSR-IV v1-3) were amplified from the brain and eyestalk ganglia cDNAs. MSR-IV v1 has >99% nt identity, whereas MSR-IV v2 has a 42-nt deletion (nt 1172–1213) in the COOH-terminal coding sequence but retains the full-length variant reading frame for the terminal seven amino acids and stop codon. MSR-IV v3 has a 312-nt deletion (nt 902–1213) that results in loss of the last two transmembrane domains but likewise retains the terminal seven amino acids and stop codon. The MSR-V clones obtained from the brain and eyestalk ganglia cDNAs contained a 1,425-nt ORF that differed from the transcriptomic sequence by a 36-nt insertion at nt 1058 that maintained the same reading frame over the final 332 nucleotides. Consensus validated sequences for all MSRs have been deposited with GenBank under accession nos. MT068477–MT068483.

**Table 3.** Matrix of percent amino acid identity/similarity between select putative Homarus americanus myosuppressin receptor proteins

	MSR-I	MSR-II-v1	MSR-IIIa	MSR-IVa	MSR-V
MSR-I		73/87	85/95	37/69	32/65
MSR-II-v1			86/96	37/69	35/68
MSR-IIIa				45/74	36/68
MSR-IVa					35/68
MSR-V					

Values are percent identity/similarity between myosuppressin receptor (MSR)-IIIa and all other receptors was calculated using only the regions of overlap, as MSR-IIIa is a COOH-terminal partial protein. Calculations are based on the proteins deduced from the transcriptomic data presented in this study.

### Differential Expression of Myosuppressin Receptors Between Neuronal Types in the Cardiac Ganglion May Underlie Physiological Response to Local Myosuppressin Release

**Fig. 9.**
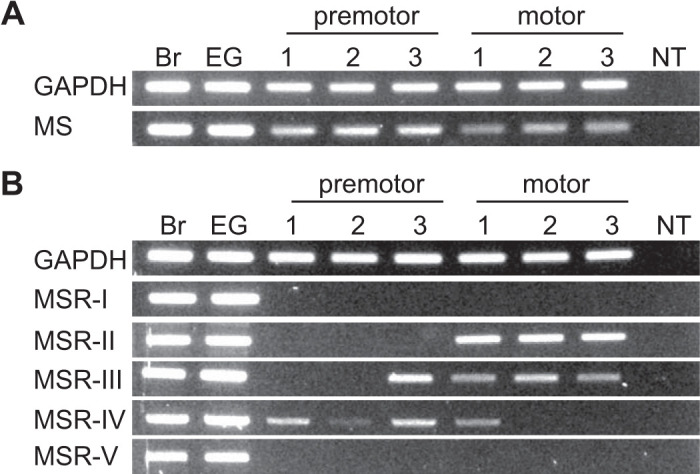
PCR confirmed the differential expression of myosuppressin and putative myosuppressin receptors (MSRs) in the premotor and motor neurons of the *Homarus americanus* cardiac ganglion (CG). RT-PCR-based amplification of the myosuppressin preprohormone (*A*) and myosuppressin receptors (*B*) are from 3 biological replicates of premotor and motor neuron cDNAs. Brain (Br) and eyestalk ganglia (EG) were included as positive controls for MSR amplification. NT corresponds to the no-template control. To confirm cDNA quality, a 500-bp fragment of *Homarus* GAPDH was amplified. Representative image corresponds to PCR products electrophoresed on 1.5% agarose gels stained with SYBR Safe.

Previous and current physiological data suggest local release of myosuppressin, which is consistent with the presence of its transcript in a CG-specific transcriptome assembly. To assess transcript expression in the two CG neuron types, we performed RT-PCR using premotor and motor neuron-specific cDNAs from multiple biological replicates as well as cDNA from brain and eyestalk ganglia. As expected, amplicons corresponding to the prepropeptide were generated from all tissues examined (Fig. 9*A*), albeit at differing intensities from the CG region-specific cDNAs, which could indicate region-specific transcriptional control.

Given the presence of five putative myosuppressin receptor transcripts in the lobster nervous system, we asked whether any were expressed in the CG, and if so, whether they were expressed differentially in mRNA isolated from the premotor and motor regions of the ganglion. First, to examine the ability of our expression profiling primers to amplify the five putative receptor transcripts, we confirmed amplification of 500-bp fragments from *H. americanus* brain and eyestalk ganglia cDNAs (Fig. 9*B*); for MSR-IV, which consists of at least three variants (i.e., MSR-IV v1-3), the primers used were designed to amplify a conserved portion of the respective variant sequences.

RT-PCR profiling of the CG region cDNAs showed that, similar to the CG transcriptomic data, transcripts encoding MSR-I and MSR-V are either not present in the two neuron types or are expressed at levels below the threshold of detection (Fig. 9*B*). Although the RT-PCR data shown here are not quantitative, we can compare relative intensities of the bands for each of the receptors, using the GAPDH housekeeping gene as a baseline. Intensities of the GAPDH bands are relatively constant across samples, suggesting that large differences between expression in the different neuronal types may reflect differences in receptor expression levels. Our results suggest that MSR-II is either motor neuron specific or that it is expressed in much higher levels in the motor neurons than in the premotor neurons. Across the six pooled replicates of the premotor neurons, MSR-II appeared at low expression levels in just one pooled sample, whereas it was prominent in all of the motor neuron samples.

Both MSR-III- and MSR-IV-encoding transcripts were present in multiple tissue replicates for both neuron types. RT-PCR results suggest that MSR-III is more abundant in the motor neurons than the premotor neurons (amplicon present in all 6 pooled motor neuron replicates vs. 2 of 6 premotor neuron replicates). In contrast, MSR-IV appeared to be expressed predominantly in the premotor neurons rather than the motor neurons (amplicon present in all 6 pooled replicates vs. 4 of 7 motor neuron replicates).

## DISCUSSION

The rhythmicity of neuronal firing observed in the lobster CG has been characterized as an exemplary model of a circuit with a grouped pacemaker configuration (Cooke 2002). In the classical view of the CG, pacemaker potentials that originate in the four small premotor neurons transmit their synchronized activity to the larger motor neurons via excitatory synapses and electrical connections to initiate motor neuron firing. The chemical and electrical coupling of these cell types is complex and raises the question as to whether cross-neuronal communication can be fully blocked.

### The Components of the CG: the Physically Decoupled Premotor and Motor Neurons

To examine the physically decoupled premotor and motor neurons, we used a physical ligature to disrupt the coordinated, in-phase bursting of the premotor and motor neurons. Previously, when the motor neurons were separated from the premotor neurons and voltage clamped, the driver potentials were shown to consist of an inward calcium current and three outward potassium currents, but no pacemaking currents were identified (Tazaki and Cooke 1986, 1990). Both motor neurons 1 and 2, when individually isolated by ligature, were able to respond to imposed depolarizing pulses with driver potentials but were quiescent in the absence of stimulation (Tazaki and Cooke 1983). However, studies demonstrated that transected *Homarus* ganglion segments containing motor neuron soma were capable of producing rhythmic bursting (Mayeri 1973; Tazaki and Cooke 1983). Mayeri (1973) noted that when bursting was present, impulses attributable to small cell axons included in the isolated nerve segment were detected in addition to large axon impulses. Tazaki and Cooke (1983) observed rhythmic spontaneous burst generation from motor neuron 3 after isolation of the ganglion segment containing its soma and proximal axons by three ligatures. However, they did not routinely monitor the extracellular activity of the isolated nerve segments; thus the potential participation of premotor neuron processes was unknown (Tazaki and Cooke 1983). While we cannot rule out the potential interaction between motor neurons and inactive premotor neuron axons that could contribute to the observed bursting, our placement of the ligature anterior to the soma of motor neuron 4 meant that no active premotor neuron axons were present (Hartline 1967), which was confirmed by extracellular recordings in which small axon impulses were absent. This suggests that the observed rhythmic bursting of the motor neurons is attributable to the intrinsic neuronal properties of one or more motor neurons. Here, we report that the premotor and motor neurons of the ganglion can establish independent bursting patterns in approximately two-thirds of preparations that have been physically decoupled by a ligature. These results suggest that the isolated motor neurons can possess intrinsic bursting properties that explain their firing pattern when decoupled from the premotor neurons.

In further characterization of the inward currents and channels underlying bursting activity of *Cancer borealis* CG neurons, Ransdell et al. (2013) identified a largely noninactivating tetrodotoxin (TTX)-sensitive current necessary for driver potential generation, which suggested the presence of a persistent sodium current, *I*_NaP_. Such currents have been shown to alter the bursting frequency and contribute to the burst generating ability of pacemaker neurons. In the DG neurons of the spiny lobster, *Panulirus interruptus*, stomatogastric ganglion (STG), the presence of *I*_NaP_ is known to be important in plateau potential generation (Elson and Selverston 1997), and a TTX-sensitive persistent sodium current has been identified in cultured *P. interruptus* STG cells (Turrigiano et al. 1995). Additional evidence in mammalian pre-Bötzinger neurons has shown that *I*_NaP_ is necessary for burst generation (Del Negro et al. 2002a, 2002b). Therefore, it is possible that the presence of a persistent sodium current in one or more of the *H. americanus* motor neurons may explain their independent bursting capability observed here. Variable expression of different currents across individuals, as seen in crabs (Ransdell et al. 2013), might underlie the variability of the responses to the ligature across animals.

Across all ligatures attempted, over 30% (15/41) of preparations did not reestablish bursting in both the premotor and motor neurons. Since we did not conduct intracellular or voltage-clamp recordings, we cannot determine the mechanism that underlies this variability. However, recent appreciation for variation in neuronal parameters such as synaptic strengths (Grashow et al. 2010; Olypher and Calabrese 2007; Wilhelm et al. 2009), conductance magnitudes (Schulz et al. 2006; Wilhelm et al. 2009), and channel activation properties (Amendola et al. 2012) that underlie identical patterned output across neurons suggest that multiple mechanisms may contribute to the CG bursting examined here. It is possible that in some CGs, the mechanisms that are important for burst generation in one or both neuron types are more resilient and able to function in isolation from the rest of the network, while in others, cross-neuronal interactions are more critical to the maintenance of bursting activity.

Ligaturing the CG did not significantly alter the duration or frequency of premotor neurons bursts but significantly increased the duration and decreased the frequency of motor neuron bursts; it also introduced a trail of leading spikes into these neurons. In the ligatured CG, the intrinsic burst duration and frequency of the premotor and motor neurons differed significantly from one another. In a two-cell model of the *Homarus* CG derived from Morris–Lecar oscillators (Morris and Lecar 1981), the two neuron types displayed different intrinsic duty cycles (Williams et al. 2013). Across model runs, the neurons were drawn toward a compromise value via synaptic coupling and predicted strong electrical coupling as a key mediator of burst synchronization between heterogeneous oscillators. The data gathered here provide further evidence for neuronal heterogeneity in this coupled network, as well as additional evidence that synchronization between the premotor and motor neurons allows the premotor neurons to drive the bursting pattern of the CG, as previously hypothesized (Hartline 1967). Moreover, the ligatured burst characteristics observed here highlight the variability in network phasing that might result from variation in strength of coupling between neuron types or in the intrinsic burst characteristics.

Separating the motor and premotor neurons with a ligature induced leading spikes in nearly all (25/26) of the ligatured motor neuron regions. Intracellular recordings would enable us to see whether or not the characteristic leading spikes are due to a depolarization of the membrane potential between driver potentials. However, this does not seem likely, as depolarization of the motor neurons in an intact ganglion leads to a higher burst frequency, whereas in the ligatured ganglion, the burst frequency decreases. In a previous study, when proctolin was applied to a motor sensitive region of the *Homarus* CG, a depolarization of the motor neurons was accompanied by an increase in burst frequency (Miller and Sullivan 1981; Sullivan and Miller 1984). This suggests that the leading spikes are due to a mechanism more complex than simple depolarization. One possibility is that when the ligature was tightened and a spike-initiating zone was removed, new spike initiation zones were established, including one that generated the leading spikes. Such establishment of new spike-initiating zones has been observed in esophageal neurons of the stomatogastric nervous system in the spiny lobster, *Jasus lalandii*. These neurons have multiple spike-initiating zones (Nagy et al. 1981); when cut, they often establish additional, new spike-initiation zones (unpublished observations).

### Myosuppressin Differentially Modulates Premotor and Motor Neurons in the CG

Here, we assessed the effects of myosuppressin on the two neuron types in the lobster CG. In both this and a previous study (Stevens et al. 2009), myosuppressin exerted clear effects on the isolated and intact CG at concentrations of 10^−6^ to 10^−8^ M. The upper ranges of the concentrations reported by Stevens et al. (2009), however, are typically associated with local rather than hormonal release (Dickinson et al. 2015, 2019a; Messinger et al. 2005). For example, when FMRFamide-like peptides were measured using a radioimmunoassay based on a FMRF-amide antibody in lobster, concentrations from 10^−11^ to 10^−10^ M were reported in circulating hemolymph (Kobierski et al. 1987). Hemolymph concentrations up to 3–4 × 10^−8^ M were reported for other circulating peptides in insects and shrimp, including vitellogenesis-inhibiting hormone and ecdysis-triggering hormone (Fastner et al. 2007; Kang et al. 2014; Zitnan et al. 1999). However, many of the effects of myosuppressin were observed only at concentrations higher than those associated with hormonal release of other neuropeptides. This, together with the fact that myosuppressin transcripts were identified in both the premotor and motor neuron regions of the CG (Fig. 9*A*), suggests that the peptide may be locally released. Since the premotor and motor neurons are connected by chemical synapses as well as electrical coupling (Cooke 2002), it is feasible that myosuppressin released from the CG itself could act as an intrinsic modulator (Katz 1995; Katz and Frost 1996) on both neuron types. Another possible source for local myosuppressin release is the descending cardioregulatory fibers that innervate the CG (reviewed in Cooke 2002; Fort et al. 2004; Fort et al. 2007a, 2007b). Because there are no antibodies specific to myosuppressin, it is not currently possible to determine whether these pathways contain and release myosuppressin. Together, these data suggest that the regulation of the heartbeat in the decapods may involve integration of information from hormonal pathways, local release from descending regulatory fibers, and release of modulators from intrinsic sources, suggesting that central regulation of the heartbeat may be considerably more complex than previously thought.

Stevens et al. (2009) examined the effects of myosuppressin in the intact lobster, on the whole heart, on the isolated CG, and on the neuromuscular junction/muscle. They determined that the global effects of myosuppressin on the cardiac neuromuscular system represent the integration of site-specific effects. At concentrations ranging from 10^−8^ M and 10^−6^ M, myosuppressin elicited a decrease in burst frequency but an increase in burst duration and contraction amplitude. The effects of myosuppressin on the intact CG in our study are consistent with these previous findings. We investigated whether these changes in burst duration and frequency are mediated by the premotor or motor neurons, particularly in the isolated CG, in which no feedback is present. While both 10^−6^ M and 10^−7^ M myosuppressin elicited changes in both ligatured neuron types, the threshold for most of the changes we observed was 10^−6^ M. The only changes elicited by 10^−7^ M myosuppressin in the ligatured ganglion were the decrease in burst frequency and increase in interburst interval in the motor neurons, suggesting that the threshold for effects of myosuppressin is lower in the motor neurons than it is in the premotor neurons. The fact that 10^−7^ M myosuppressin is sufficient to elicit frequency changes in both neuronal types when the CG is intact but only in the motor neurons when ligatured suggests that the motor neurons play an important role in determining the burst frequency and interburst interval of the intact CG firing pattern, at least in the presence of low concentrations of myosuppressin. Myosuppressin might cause these changes by activating pathways that hyperpolarize the motor neurons, as was seen previously (Stevens et al. 2009), resulting in a decrease in burst frequency. Alternatively, it could also activate pathways that specifically affect the pacemaker potential, resulting in a slower rate of depolarization to the next burst.

In the intact CG, 10^−6^ M myosuppressin elicited an increase in burst duration that was not observed with 10^−7^ M peptide application. While neither concentration tested here elicited an increase in burst duration in the ligatured motor neurons, 10^−6^ M myosuppressin was capable of eliciting an increase in the burst duration of the ligatured premotor neurons. These data suggest that the premotor neurons are responsible for the increase in burst duration observed in the intact ganglion in response to 10^−6^ M myosuppressin.

The differential modulation by myosuppressin of the premotor and motor neurons suggests that the peptide may target different channels and currents in the two neuron types. In their characterization of the neurons of the *Homarus* CG, Tazaki and Cooke (1983) highlighted the role of driver potentials and a pacemaker potential in impulse generation. Given that myosuppressin appears to primarily alter the burst frequency of the isolated motor neurons, it is likely that myosuppressin targets a pacemaking potential in the motor neurons, such as the persistent sodium current previously identified in crab CG (Ransdell et al. 2013). Myosuppressin largely altered the burst duration of the isolated premotor neurons, suggesting that the peptide may target a different current or currents, specifically those implicated in the driver potentials, which underlie the bursts of action potentials in CG neurons, generated in this neuron type. If so, it appears that the sum of these interactions is what drives the changes in the patterned output of the intact CG.

Nonetheless, we cannot rule out the possibility that the application of myosuppressin to ligatured preparations elicited effects that crossed the boundary that the ligature creates between neuronal types. For example, if myosuppressin receptors are present in the regions of the premotor neurons that remain in the motor neuron region after ligature, those regions of the premotor neurons could be activated and influence the motor neurons, and vice versa. The two neuronal types are tightly interconnected so that complete separation of the neuron types is impossible.

Although the motor neurons have previously been considered a relatively homogenous set of neurons due to their tight electrical coupling, the data presented here do not provide sufficient evidence to determine whether myosuppressin has the same effect on all motor neurons. Intracellular recordings from the ligatured ganglion would provide further information about the action of myosuppressin on individual motor neurons. The appearance of the leading spikes, which appear to be singular action potentials generated from a single neuron, raises the possibility that individual motor neurons may serve distinct roles in the pattern generator output.

### Differential Myosuppressin Receptor Distribution May Contribute to Differential Modulation of Neuron Types

Recent advances in genomics/transcriptomics have enabled novel peptide identification in a variety of animals, including crustaceans, and have prompted investigation of the ability of neuropeptides to modulate rhythmic motor behaviors (Blitz 2017; Christie 2014a, 2014b, 2014c; Christie et al. 2010a, 2011; Dickinson et al. 2016). However, published studies that examine the extent to which the differences in physiological effects are related to differential receptor expression at the neuronal level are sparse. In the STG of *C. borealis*, CCAP receptor expression was assessed, and expression levels differed significantly between specific neuron types, in correlation with responses to the peptide (Garcia et al. 2015). Here, we examined the expression of five myosuppressin receptor transcripts (MSR-I–V) and found apparent differential expression across the premotor and motor neurons.

The predominant expression of MSR-II and MSR-III transcripts in the motor neurons relative to the premotor neurons (Fig. 9*B*) is consistent with a potential role for MSR-II and MSR-III functionality in the decreased frequency that defines the bursting pattern of these neurons when ligatured. If MSR-II or MSR-III plays a similar role in all CG neurons, this would be consistent with the observation that the premotor neurons exhibit smaller changes in frequency with myosuppressin application, as MSR-II appears to have lower expression in the premotor neurons. Conversely, MSR-IV, which appears to be expressed at lower levels in the motor neurons, may primarily alter the driver potentials that define the burst duration of the coordinated ganglionic output. However, since mRNA can be trafficked throughout neurons, we cannot rule out the possibility that local mRNAs from premotor neuron presynaptic terminals or axons were collected with the motor neuron cell body tissue, or vice versa. Due to the intertwining of the axonal terminals and cell bodies, any separation of these neuron types is imperfect; in the procedure employed here, ganglionic tissue was divided to separate the premotor and motor neuron cell bodies, but terminals and axons were not fully separated.

### Conclusions

The *Homarus americanus* cardiac neuromuscular system is a CPG-effector system that has been well studied at the level of the whole heart, the isolated CG (the CPG), and the isolated muscle (effector system). The patterned output can be modulated in response to an expansive class of neuropeptides, yet there remain few investigations of the CG at the level of the individual neuron types. In this work, we demonstrate that the premotor and motor neurons establish separate bursting patterns when decoupled by a physical ligature and that their independent modulation by the neuropeptide myosuppressin may result from a differential distribution of myosuppressin receptors across neuron types.

Our results thus extend the literature on the *Homarus* CG, providing insight into the cellular components of the CPG. Future work addressing the variable mechanisms that may underlie the bursting capabilities of the separated neurons observed here would further elucidate the role of each neuron type in producing coordinated ganglionic output.

## GRANTS

Funding was provided by National Science Foundation Grants IOS-1353023 and IOS-1354567, National Institute of General Medical Sciences Institutional Development Award (IDeA) P20GM103423, U.S. Department of Agriculture (USDA) Current Research Information System (CRIS) Project 2020-22620-022-00D, the Cades Foundation, the Henry L and Grace Doherty Coastal Studies Research Fellowship, the Arnold and Mabel Beckman Foundation, and the Paller Fund of Bowdoin College.

## DISCLAIMERS

Mention of trade names/commercial products in this article is solely for the purpose of providing specific information and does not imply recommendation/endorsement by the U.S. Department of Agriculture. USDA is an equal opportunity provider/employer.

## DISCLOSURES

No conflicts of interest, financial or otherwise, are declared by the authors.

## AUTHOR CONTRIBUTIONS

E.R.O., A.E.C., and P.S.D. conceived and designed research; E.R.O., M.E.S., J.J.H., A.E.C., and P.S.D. performed experiments; E.R.O., J.J.H., A.E.C., and P.S.D. analyzed data; E.R.O., J.J.H., A.E.C., and P.S.D. interpreted results of experiments; E.R.O., J.J.H., A.E.C., and P.S.D. prepared figures; E.R.O., J.J.H., A.E.C., and P.S.D. drafted manuscript; E.R.O., M.E.S., J.J.H., A.E.C., and P.S.D. edited and revised manuscript; E.R.O., M.E.S., J.J.H., A.E.C., and P.S.D. approved final version of manuscript.
